# Association between socioeconomic and motherhood characteristics with receiving community-based treatment services among justice-involved young female drug users: a retrospective cohort study in Taiwan

**DOI:** 10.1186/s12954-024-01010-0

**Published:** 2024-06-05

**Authors:** Chuan-Yu Chen, Tan-Wen Hsieh, Wenmay Rei, Cheng-Hsiung Huang, Sheng-Chang Wang

**Affiliations:** 1 Institute of Public Health, National Yang Ming Chiao Tung University, Medical Building II, No. 155, Sec. 2, Linong Street, Taipei, 112 Taiwan; 2 Center for Neuropsychiatric Research, National Health Research Institutes, Zhunan, Taiwan; 3 Taiwan High Prosecutors Office, Taipei, Taiwan

**Keywords:** Women, Deferred prosecution, Childbearing, Amphetamine, Alternative to incarceration

## Abstract

**Background:**

Drug-involved individuals who contact treatment services in Taiwan are mostly driven by criminal justice systems either as an alternative or adjunct to criminal sanctions for a drug offence. With a focus on justice-involved young female drug users, the present study examines the extent to which socioeconomic and motherhood characteristics are associated with receiving deferred prosecution, a scheme diverting drug offenders to community-based addiction treatment.

**Methods:**

We identified a cohort of 5869 women under the age of 30 arrested for using Schedule II drugs (primarily amphetamine-like stimulants) from the 2011–2017 National Police Criminal Records in Taiwan. Information concerning socioeconomic characteristics, pregnancy and live birth history, and deferred prosecution was obtained through linkage with the 2006–2019 National Health Insurance, birth registration, and deferred prosecution datasets. Multinomial logistic regression was used to evaluate the association with stratification by recidivism status.

**Results:**

Within six months of arrest, 21% of first-time offenders (n = 2645) received deferred prosecution and 23% received correction-based rehabilitation; the corresponding estimates for recidivists (n = 3224) were 6% and 15%, respectively. Among first-time offenders, low/unstable income was associated with lower odds of deferred prosecution (adjusted odds ratio [aOR] = 0.71; 95% CI: 0.58, 0.88). For recidivists, those with low/unstable income (aOR = 1.58) or unemployment (aOR = 1.58) had higher odds of correction-based rehabilitation; being pregnant at arrest was linked with reduced odds of deferred prosecution (aOR = 0.31, 95% CI: 0.13, 0.71) and correction-based rehabilitation (aOR = 0.50, 95% CI: 0.32, 0.77).

**Conclusions:**

For the young women arrested for drug offences, disadvantaged socioeconomic conditions were generally unfavored by the diversion to treatment in the community. Childbearing upon arrest may lower not only the odds of receiving medical treatment but also correctional intervention. The criminal prosecution policy and process should be informed by female drug offenders’ need for treatment and recovery.

**Supplementary Information:**

The online version contains supplementary material available at 10.1186/s12954-024-01010-0.

## Background

Individuals affected by drug-use disorders increased by 34% from 1990 to 2017 [1], and over 280 million individuals aged 15–64 have used at least one type of illegal drug within the last 12 months worldwide [2]. Drug use alone has been responsible for 1.3% of the burden of diseases and injuries globally—an estimated 31.8 million disability-adjusted life-years (DALYs) in 2016; and the corresponding percentage among young people was two to three times higher [3, 4]. Because the use of certain drugs per se is still criminalized in most societies, drug use also contributes to an intense burden on the criminal justice system. In high-income countries, almost one in three male and one in two female newly incarcerated individuals experienced drug-use disorders in the previous 12 months [5]; one-fourth of the adults on probation and one-third on parole were imprisoned for a drug offence in the United States [6]. In low- and middle-income countries, nearly one in three prisoners has ever experienced drug-use disorders; for women, the estimates fell between 25 and 50% [7].

Empirical evidence generally supports that, for non-violent minor drug offences, conviction and incarceration alone not only have limited effectiveness in deterring drug use but also exert harm on drug users, families, and the community [8, 9]. As the disease model of addiction has been gradually embraced over recent decades, several alternatives to incarceration have been implemented to tackle drug-use problems, including community supervision and treatment, drug courts, encouraging treatment, and drug treatment in prison [8, 10, 11]. The importance of self-determination and internal motivation for positive treatment outcomes, scaling-up, and ensuring access to effective and quality treatment has often been listed as the highest priority for promoting voluntary help-seeking. Nonetheless, some forms of coercive treatment regimens remain common and have even increased in recent years in some Asian countries [12, 13]. Drug-involved individuals who contact treatment services were driven by criminal justice systems either as an alternative or adjunct to criminal sanctions for a drug offence. Although findings concerning the effectiveness of coerced treatment are ambiguous, and other ethical concerns have been consistently raised [12, 14–16], mandatory treatment undoubtedly provides a means to ensure access to treatment for those who would otherwise not enter treatment [12].

Although men have outnumbered women in the prevalence of drug use and disorders, such gender differences have been narrowing in young cohorts and with certain drugs [17–19]. Cumulative evidence has demonstrated that women and men have distinct clinical manifestations and pathways for addiction and crime [18, 20–22]. For example, once having exposure to or initiating drugs, women not only transitioned into regular use or clinical disorder more rapidly than men but also endorsed a higher risk of developing severe medical, functional, and social impairment [18–20]. Meanwhile, although women were less likely to be sentenced to prison and received shorter sentences than men for drug offences [21], individuals affected by drug use or disorder were often overrepresented in the incarcerated population for women (~ 60%)—1.5–2-fold higher than men [5, 8, 23]. Upon conviction and incarceration, women appeared more vulnerable to collateral consequences, such as housing challenges, decreased employment opportunities, reduced access to welfare and benefits, and intergenerational criminality [11, 24, 25]. In the context of the rising number of drug-using women and the mounting societal burden, there is an urgent need to understand gender-specific features in the need for treatment and recovery.

For women in Taiwan, the use of illegal drugs has been perceived as “double deviance,” given that it is against both the formally enacted laws and the social norms toward women/mothers (i.e., gender roles) [26]. The road to treatment and recovery is often impeded by barriers related to stigma, a lack of gender-tailored treatment or gender-responsive services, financial disadvantages, and childcare or custody issues [8, 27, 28]. Although emerging literature has begun to focus on the diversion of drug-related offenders from the criminal justice system and the potential effectiveness of gender-specific programs [29, 30], much less is known about factors that affect opportunities to engage in community-based treatment among justice-involved women—specifically whether motherhood characteristics may play a role in the diversion or referral process.

Nonmedical use of amphetamine-like stimulants has emerged as an important public health issue worldwide, especially in high-income North American countries and Australasia [12, 31, 32]. In Taiwan, partly due to lower prices and ready availability, the use of methamphetamine and amphetamine has been on a steady rise over the past two decades, especially among young people and women [33, 34]. Against the background that deferred prosecution has been progressively utilized as a mechanism to gain access to evidence-based care in the community, we retrospectively ascertained a cohort of young women who were arrested for the consumption of Schedule II illegal drugs (primarily amphetamine-like stimulants). The main objective was to describe the status of prosecutorial decisions for treatment diversion and explore the associated factors. Beyond legal and addiction variables, we direct attention to whether socioeconomic status and motherhood characteristics may affect women receiving community-based or correction-based addiction services.

## Methods

### Study setting

Since the enactment of the Narcotics Hazard Prevention Act in 1998, Taiwan has officially viewed users of scheduled, controlled substances (i.e., illegal drugs) not only as criminals but also as patients [35]. Many medical practitioners have taken a similar progressive stand on the issue of reducing (illegal) drug use and related harm. Even so, legal mandates persist as the primary mechanism by which illegal drug users engage in addiction consultation or treatment. At present, health-oriented addiction services offered to justice-involved drug users have two models [33, 35]. The first is an abstinence-oriented compulsory abstention/rehabilitation program exclusively based in correctional facilities. For first-time drug offenders, up to two months of observation is ordered for detoxication, which can be extended for 6–12 months of rehabilitation when a high tendency toward recidivism is indicated. Drug education and group consultation are among the approaches provided in correctional facilities, with no access to addiction treatment medications (e.g., methadone). The second model is treatment implemented through a deferred-prosecution procedure that mandates offenders engage in self-funded community-based addiction treatment [33, 36]. Upon receiving deferred prosecution, drug offenders should attend community-based treatment for one year and periodically be monitored for compliance (e.g., urine testing); after successful completion of the treatment, the original charges can be dismissed (or expunged). Deferred prosecution offers drug offenders the opportunity not only to avoid consequences linked with having a criminal record (e.g., reduced employment opportunity) and disrupted involvement in family activities but also to ensure their access to quality healthcare in communities.

Until late 2008, when the Deferred Prosecution with Condition to Complete the Addiction Treatment (DPCCAT) was implemented with a major focus on Schedule I drugs (mostly heroin), compulsory abstention/rehabilitation programs were the only “treatment” option for drug offenders; in 2013, this was scaled up for offences involving Schedule II drugs (primarily involving amphetamine-like stimulants). For Schedule II drug offences, recidivists generally receive incarceration, with a maximum three-year fixed term. Female offenders who are more than five months pregnant or gave birth less than two months prior cannot be incarcerated; the agency of corrections may approve out-of-prison preparation for birth and postpartum recovery on bail.

### Study design and population

Our study utilized several national administrative datasets concerning healthcare, social welfare, and criminal justice services to the individuals involved in illegal drug-related activities compiled in the Datasets of Drug Abuse Intervention managed by the Ministry of Health and Welfare in Taiwan, including the Police Criminal Record Processing System and Household Registration Records from the Ministry of the Interior; the Prison Entry and Exit Registers and the DPCCAT dataset from the Ministry of Justice; and the Birth Reporting System (BRS), the Death Registration System, and the National Health Insurance Database from the Ministry of Health and Welfare.

Considering that emerging adulthood is the critical period for intervention on substance use disorders and peak reproductive years [37], the present study is therefore restricted to women in their late teens and twenties. Through the Police Criminal Record Processing System, we initially identified a cohort of 9653 women who have been arrested for the use of Schedule II drugs (e.g., amphetamine, methamphetamine, 3,4-Methylenedioxymethamphetamine [MDMA]) at least once during the ages of 18–29 during the calendar years of 2011–2017. For the women who had two or more Schedule II drug use related arrest records (39.2%), the present study uses the most recent record as the index arrest, resulting in an analytic sample of 5869. All the data linkages and analyses were performed through the encrypted identification number in the Health and Welfare Data Science Center. To ensure de-identification, any statistics with fewer than three observations were not allowed to be presented.

### Measures

In the present study, the outcome variable—the status of a prosecutorial decision within six months of the index arrest for the use of Schedule II drugs—was obtained from the Prison Entry and Exit Registers, the DPCCAT, and the Death Registration System during the years 2011–2017. For the variables of interest, we ascertained pregnancy status upon arrest from outpatient and inpatient care claim data from the 2010–2019 National Health Insurance Database (ICD-9 codes 632, 634–638, 779.6; ICD-10 codes O02.1, O03–O07, Z33.2), and the BRS (both live and stillbirths). Also, the records of having live births within five years before the index arrest were retrieved from the 2006–2019 BRS, serving as a proxy for having one or more young children upon the index arrest. Age was categorized into two groups (18–24 and 25–29 years) to reflect its nonlinear relationship with the odds of a prosecutorial decision. For pre-arrest socioeconomic characteristics, we retrieved educational attainment (categorized as elementary school, middle school, and high school or above) and marital status (categorized as single, married, and divorced/widowed) through the Household Registration Records. Household income (i.e., low income [poverty], unstable/no income, medium income, and high income) was assessed through the National Health Insurance Dataset.

Additionally, for drug-related criminal justice indicators, we obtained the primary drugs of involvement for the index arrest (i.e., type of Schedule II drug) and type of drug offence (e.g., use, nonuse, or both) from the Police Criminal Record Processing System. Correctional rehabilitation and drug-related incarceration in the five years before the index arrest was validated through the Prison Entry and Exit Registers. History of drug offences taking place more than five years before the index arrest and after the age of 18 was also retrieved accordingly.

### Statistical analyses

Since the prosecutorial decision for deferred prosecution was prioritized for first-time drug offenders, we therefore carried out the analyses with stratification by recidivist status. Thus, first-time offenders were defined as young women without drug-related arrest, correction-based treatment, or incarceration within the five years before the index arrest. In comparison, recidivist offenders were defined as having had any drug offence in the five years before the index arrest. Descriptive analyses were first utilized to characterize our participants’ socioeconomic characteristics, drug-related-crime indicators, the experience of reproductive outcomes, and the status of receiving deferred prosecution in the young women arrested for Schedule II drug use in the period from 2011 to 2017.

**Fig. 1:**
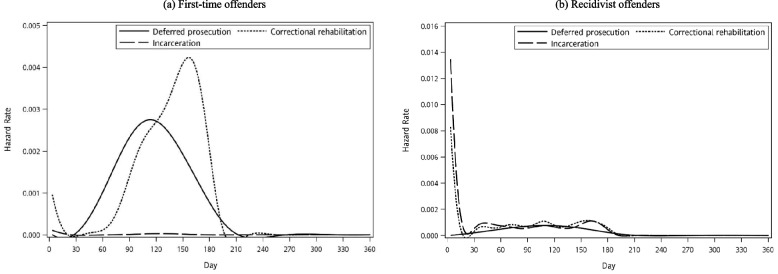
Hazard rates of deferred prosecution, correctional rehabilitation, and incarceration over twelve months after the index arrest (The figures are categorized into two groups based on prior drug-related arrests: first-time offenders and recidivist offenders)

Next, we plotted the smoothed hazard rate of receiving deferred prosecution, correctional rehabilitation, and incarceration month by month from the date of the index arrest to the end of the twelfth month, stratified by recidivism (see Fig. 1). Considering the motivation for treatment and the hazard rate of prosecutorial action, the present study then focused on the first six months after arrest. Next, we turned to multinomial logistic regression to model the association linking socioeconomic and motherhood characteristics with the status of prosecutorial decisions/implementation. For first-time drug offenders, the outcome response included deferred prosecution, correctional rehabilitation, and awaiting a decision/execution; for recidivists, incarceration was a fourth response. In this series of analyses, awaiting a decision/execution was the reference outcome. Model I examined the role of socioeconomic and motherhood characteristics, one by one, with simultaneous adjustment for drug-related crime factors. Next, all socioeconomic and motherhood characteristics were entered into Model II. The sample size should allow us to examine the association with precise estimates by the rule of ten events per variable [38]. Finally, because more than half were awaiting status and because receiving deferred prosecution is the focal indicator for access to community-based treatment, we also ran a series of sensitivity analyses to evaluate how socioeconomic and motherhood characteristics are linked with the time to receive deferred prosecution by taking the censoring of data into account (see Appendix materials). All analyses were performed by SAS 9.4 (SAS Institute Inc., Cary, NC, USA).

## Results

As shown in Table 1, disadvantaged socioeconomic conditions appeared more prevalent among the recidivist offenders, such as unstable low income (65.3% vs. 76.4%), lower educational attainment (elementary school: 7.0% vs. 10.6%), and unemployment (38.7% vs. 50.1%). Approximately 8% of recidivist offenders were pregnant upon arrest, and 27% had at least one young child, significantly higher than the corresponding 4.1 and 17.3% among the first-time offenders. First-time drug offenders were found to be less likely to be arrested for concurrent engagement in Schedule I drugs (2.9% vs. 10.7%). Within six months after being arrested, 20.9% of first-time drug offenders and 6.0% of former offenders received deferred prosecution, and the estimates for correctional rehabilitation were 22.8% and 15.1%, respectively (*p* < 0.0001).

**Table 1:** Characteristics of Schedule II female drug offenders under age 30, by prior 5-year drug-related arrest^a^ (N = 5869)

Variables	Drug offenders		Illegal drug offence in prior 5 years				*p*
			First-time offenders		Recidivist offenders		
	(n = 5869)		(n = 2645)		(n = 3224)		
	n	%	n	%	n	%	
**Individual characteristics**							
Age (years)							
25–29	2910	49.6	1054	39.9	1856	57.6	< 0.0001
18–24	2959	50.4	1591	60.1	1368	42.4	
Marital status^b^							
Single	3935	67.1	1929	72.9	2006	62.2	< 0.0001
Married	893	15.2	320	12.1	573	17.8	
Divorced or widowed	1033	17.6	390	14.7	643	19.9	
Educational attainment^b^							
Senior high school or above	1455	24.8	753	28.5	702	21.8	< 0.0001
Junior high school	3820	65.1	1666	63.0	2154	66.8	
Elementary school	527	9.0	185	7.0	342	10.6	
Employment status upon arrest^b,c^							
Unemployed	2639	45.0	1024	38.7	1615	50.1	< 0.0001
Student	150	2.6	108	4.1	42	1.3	
Employed	3045	51.9	1496	56.6	1549	48.1	
Income level (by insurance status)^b^							
Low income (poverty)	146	2.5	68	2.6	78	2.4	< 0.0001
Unstable/no income	4041	68.9	1657	62.7	2384	74.0	
Medium income	1421	24.2	806	30.5	615	19.1	
High income	130	2.2	80	3.0	50	1.6	
Being pregnant upon arrest							
No	5516	94.0	2538	95.9	2978	92.4	< 0.0001
Yes	353	6.0	107	4.1	246	7.6	
Having a young child							
None	4518	77.0	2177	82.3	2341	72.6	< 0.0001
1	1015	17.3	368	13.9	647	20.1	
2	283	4.8	89	3.4	194	6.0	
≥ 3	53	0.9	11	0.4	42	1.3	
Drug offence in prior five years							
None	2645	45.1	2645	100.0	0	0	< 0.0001
Drug use only	1410	24.0	0	0.0	1410	43.8	
Non-drug use only	469	8.0	0	0.0	469	14.5	
Both	1345	22.9	0	0.0	1345	41.7	
Drug offence more than five years before^d^							
No	5052	86.1	2520	95.3	2532	78.5	< 0.0001
Yes	817	13.9	125	4.7	692	21.5	
**Characteristics of the index arrest**							
Schedule II drug type							
Amphetamine	4191	71.4	1581	59.8	2610	80.9	< 0.0001
Methamphetamine	519	8.8	180	6.8	339	10.5	
MDMA/MMDA	870	14.8	696	26.3	174	5.4	
Cannabis	79	1.4	69	2.6	10	0.3	
Other (e.g., GHB, Codeine)	58	1.0	46	1.7	12	0.4	
Two or more	152	2.6	73	2.8	79	2.5	
Involvement in Schedule I drugs							
Heroin	365	6.2	72	2.7	293	9.1	< 0.0001
Opioid/morphine	59	1.0	6	0.2	53	1.6	
None	5445	92.8	2567	97.1	2878	89.3	
Drug offence other than use (e.g., possession)							
No	5455	92.9	2461	93.0	2994	92.9	0.7916
Yes	414	7.1	184	7.0	230	7.1	
**Prosecutorial decision**							
Awaiting	3381	57.6	1480	56.0	1901	59.0	< 0.0001
Receiving deferred prosecution	746	12.7	552	20.9	194	6.0	
Correctional rehabilitation	1089	18.6	602	22.8	487	15.1	
Incarceration	634	10.8	6	0.2	628	19.5	
Death	19	0.3	5	0.2	14	0.4	

^a^Defined by zero records in the Police Criminal Record Processing System and Prison Entry and Exit registers

^b^Numbers may not sum to 100% due to missingness (< 3%)

^c^Employment status was obtained from the Police Criminal Record Processing System

^d^Only in adulthood

Figure 1 shows the instantaneous hazard to enforcing deferred prosecution, correctional rehabilitation, and incarceration within twelve months of being arrested by the prior five-year history of a drug offence. For first-time offenders, the hazard rate of deferred prosecution sharply increased after the first month and peaked around the fourth month (solid line), and the hazard rate of correctional rehabilitation peaked about two months later (dotted line). As to the recidivists, the highest hazard rates of correctional rehabilitation and incarceration (broken line) both emerged within the first month of being arrested, whereas the hazard rate for deferred prosecution was relatively flat over the first six months.

Among first-time drug offenders (Table 2), univariable analyses showed that low/unstable income was linked with reduced deferred prosecution (relative to awaiting prosecutorial decision) within the first six months of being arrested (odds ratio [OR] = 0.70) and having at least one child may lower such odds by 25%. Having low/unstable family income may lower the odds of correction-based correctional rehabilitation by 25%, and having at least one young child and being pregnant upon arrest may lower the odds by 31% and 47%, respectively. With all listed variables adjusted, women with low/unstable income were less likely to receive deferred prosecution (Model II: aOR = 0.71; 95% CI: 0.58, 0.88) and correctional rehabilitation (Model II: aOR = 0.77; 95% CI: 0.63, 0.95). The low/unstable family income-related reduced hazard to receive deferred prosecution is also found in the survival analyses (see Additional file 1: Appendix Table S1: adjusted hazard ratio [aHR] = 0.84; 95% CI: 0.72, 0.98).

**Table 2:** Characteristics in relation to prosecutorial decisions in first-time female drug offenders (n = 2634)^a^

Variables	Deferred prosecution							Correctional rehabilitation					
	Univariate			Model I^c^		Model II^d^		Univariate		Model I^c^		Model II^d^	
	OR	95% CI		aOR^b^	95% CI	aOR^b^	95% CI	OR	95% CI	aOR^b^	95% CI	aOR^b^	95% CI
Age (ref: 25–29 years)													
18–24	1.04	(0.85, 1.27)		0.95	(0.77, 1.16)	0.91	(0.73, 1.13)	1.17	(0.96, 1.42)	1.07	(0.88, 1.32)	1.04	(0.84, 1.30)
Marital status (ref: single)													
Married	0.84	(0.62, 1.14)		0.86	(0.63, 1.16)	0.95	(0.67, 1.35)	0.71*	(0.52, 0.97)	0.72*	(0.53, 0.98)	0.89	(0.63, 1.27)
Divorced or widowed	0.86	(0.64, 1.14)		0.88	(0.66, 1.17)	0.99	(0.71, 1.37)	0.96	(0.74, 1.26)	0.99	(0.75, 1.29)	1.19	(0.87, 1.61)
Educational attainment (ref: senior high school or above)													
Junior high school	0.92	(0.74 1.14)		0.92	(0.74, 1.14)	0.97	(0.78, 1.22)	0.98	(0.79, 1.20)	0.98	(0.79, 1.21)	1.00	(0.80, 1.24)
Employment status (ref: employed)													
Unemployed/student	0.91	(0.75, 1.11)		0.91	(0.74, 1.11)	0.93	(0.76, 1.14)	1.09	(0.90, 1.32)	1.08	(0.89, 1.31)	1.14	(0.94, 1.38)
Income level (ref: medium/high income)													
Low/unstable income	0.70***	(0.57, 0.86)		0.71**	(0.58, 0.87)	0.71**	(0.58, 0.88)	0.75**	(0.62, 0.92)	0.76**	(0.62 0.93)	0.77*	(0.63, 0.95)
Having one or more young children (ref: none)													
Yes	0.75*	(0.58, 0.98)		0.75*	(0.58, 0.98)	0.79	(0.58, 1.08)	0.69**	(0.53, 0.89)	0.69**	(0.53, 0.90)	0.71*	(0.52, 0.96)
Being pregnant upon arrest (ref: no)													
Yes	0.94	(0.59, 1.52)		0.95	(0.59, 1.53)	1.04	(0.64, 1.69)	0.53*	(0.30, 0.94)	0.53*	(0.30, 0.94)	0.58	(0.33, 1.04)
Schedule II drugs involvement (ref: two or more)													
One only	0.91	(0.50, 1.64)				0.83	(0.45, 1.50)	0.88	(0.50, 1.55)			0.84	(0.47, 1.49)
Schedule I drug involvement (ref: yes)													
No	1.10	(0.61, 1.99)				0.89	(0.48, 1.64)	0.99	(0.57, 1.74)			0.84	(0.47, 1.49)
Drug offence other than use (ref: no)													
Yes	0.33***	(0.20,	0.55)			0.32***	(0.19, 0.54)	0.62*	(0.42, 0.92)			0.61*	(0.41, 0.91)
Drug offence more than five years before (ref: yes)													
No	1.69*	(1.02, 2.81)				1.78*	(1.05, 3.03)	2.07**	(1.22, 3.52)			2.00*	(1.16, 3.48)

^a^Multinomial regression model, with awaiting decision/execution (reference), deferred prosecution, and correctional rehabilitation as three outcome responses

^b^aOR: adjusted odds ratio

^c^Model I presents the estimates, one by one, with adjustment for drug-offence characteristics

^d^Model II includes all listed sociodemographic, motherhood, and drug-offence characteristics

^*^p < 0.05, **p < 0 .01, ***p < 0.001

For recidivist drug offenders (Table 3), univariable analyses revealed that worse-off socioeconomic condition was not associated with deferred prosecution but with increased correction-based rehabilitation (e.g., unemployment, OR = 1.49; 95% CI: 1.22, 1.83) and incarceration (e.g., unstable/low income, OR = 1.91; 95% CI: 1.48, 2.46). Motherhood characteristics—having at least one young child (OR = 0.66; 95% CI: 0.46, 0.96) and being pregnant upon arrest (OR = 0.29, 95% CI: 0.13, 0.67) appeared to be the only non-legal factors linked with deferred prosecution. In addition, being pregnant upon arrest was also linked with reduced correctional intervention—both rehabilitation (OR = 0.52) and incarceration (OR = 0.41). With statistical adjustment for listed variables (Model II), women with unstable/low income were found more likely to receive correctional rehabilitation (aOR = 1.58, 95% CI: 1.22, 2.06) and incarceration (aOR = 1.54; 95% CI: 1.17, 2.01) within six months of arrest. Finally, childbearing upon arrest was linked with a lowered odds of receiving any prosecutorial decision, including deferred prosecution (aOR = 0.31; 95% CI: 0.13, 0.71) and correctional rehabilitation (aOR = 0.50; 95% CI: 0.32, 0.77). The childbearing-related reduced risk of receiving deferred prosecution is more salient in the survival analysis approach (see Additional file 1: Appendix Table S2: aHR = 0.43; 95% CI: 0.22, 0.85); having at least one child may lower the hazard of receiving deferred prosecution by 30%.

**Table 3:** Characteristics in relation to prosecutorial decisions in recidivist female drug offenders (n = 3210)^a^

Variables	Deferred prosecution						Correctional rehabilitation					
	Univariate		Model I^c^		Model II^d^		Univariate		Model I^c^		Model II^d^	
	OR	95% CI	aOR^b^	95% CI	aOR^b^	95% CI	OR	95% CI	aOR^b^	95% CI	aOR^b^	95% CI
Age (ref: 25–29 years)												
18–24	1.29	(0.96, 1.73)	1.28	(0.95, 1.73)	1.30	(0.95, 1.80)	1.55***	(1.27, 1.89)	1.52***	(1.24, 1.86)	1.59***	(1.28, 1.97)
Marital status (ref: single)												
Married	0.82	(0.54, 1.24)	0.82	(0.54, 1.25)	1.13	(0.72, 1.80)	0.82	(0.63, 1.09)	0.83	(0.63, 1.09)	0.95	(0.69, 1.30)
Divorced or widowed	0.92	(0.63, 1.35)	0.92	(0.63, 1.34)	1.17	(0.77, 1.78)	0.83	(0.64, 1.08)	0.84	(0.64, 1.09)	0.97	(0.72, 1.29)
Educational attainment (ref: senior high school or above)												
Junior high school	0.83	(0.59, 1.17)	0.86	(0.61, 1.22)	0.83	(0.58, 1.18)	0.93	(0.74, 1.18)	0.95	(0.75, 1.20)	0.88	(0.69 1.12)
Employment status (ref: employed)												
Unemployed/student	1.06	(0.79, 1.43)	1.07	(0.79, 1.44)	1.15	(0.85, 1.56)	1.49***	(1.22, 1.83)	1.52***	(1.24, 1.86)	1.58***	(1.28 1.94)
Income level (ref: medium/high income)												
Low/unstable income	0.88	(0.62, 1.23)	0.92	(0.65, 1.29)	1.02	(0.72, 1.44)	1.38*	(1.07, 1.78)	1.42**	(1.10, 1.84)	1.58***	(1.22 2.06)
Having one or more young children (ref: no)												
Yes	0.66*	(0.46, 0.96)	0.68*	(0.47, 0.98)	0.68	(0.45, 1.02)	0.90	(0.72, 1.13)	0.92	(0.74, 1.16)	0.91	(0.70, 1.18)
Being pregnant upon arrest (ref: no)												
Yes	0.29**	(0.13, 0.67)	0.30**	(0.13, 0.70)	0.31**	(0.13, 0.71)	0.52**	(0.34, 0.79)	0.53**	(0.35, 0.81)	0.50**	(0.32, 0.77)
Schedule II drugs involvement (ref: two or more)												
One only	0.90	(0.38, 2.12)			0.91	(0.38, 2.18)	3.46*	(1.25, 9.59)			3.26*	(1.16, 9.11)
Schedule I drug involvement (ref: yes)												
No	0.99	(0.59, 1.67)			0.82	(0.48, 1.42)	1.40	(0.94, 2.08)			1.19	(0.79, 1.80)
Drug offence other than use (ref: no)												
Yes	0.99	(0.57, 1.71)			1.03	(0.59, 1.82)	0.61*	(0.39, 0.95)			0.68	(0.43, 1.07)
Type of drug offence in prior 5 years (ref: both)												
Drug use only	2.11***	(1.46, 3.04)			2.06***	(1.43, 2.99)	1.37**	(1.09, 1.72)			1.32*	(1.05, 1.67)
Other than drug use	2.42***	(1.55, 3.80)			2.28***	(1.45, 3.60)	1.88***	(1.42, 2.51)			1.81***	(1.35, 2.42)

Variables	Incarceration					
	Univariate		Model I^c^		Model II^d^	
	OR	95% CI	aOR^b^	95% CI	aOR^b^	95% CI
Age (ref: 25–29 years)						
18–24	0.48***	(0.40, 0.59)	0.54***	(0.44, 0.66)	0.55***	(0.44, 0.68)
Marital status (ref: single)						
Married	1.09	(0.86, 1.38)	1.01	(0.79, 1.30)	0.91	(0.69, 1.21)
Divorced or widowed	1.05	(0.84, 1.33)	1.02	(0.80, 1.30)	0.85	(0.66, 1.11)
Educational attainment (ref: senior high school or above)						
Junior high school	1.30*	(1.03, 1.63)	1.19	(0.94, 1.51)	1.25	(0.98, 1.59)
Employment status (ref: employed)						
Unemployed/student	1.66***	(1.38, 1.99)	1.49***	(1.23, 1.81)	1.55***	(1.28, 1.89)
Income level (ref: medium/high income)						
Low/unstable income	1.91***	(1.48, 2.46)	1.70***	(1.31, 2.21)	1.54**	(1.17, 2.01)
Having one or more young children (ref: no)						
Yes	1.16	(0.95, 1.41)	1.06	(0.86, 1.30)	1.08	(0.86, 1.37)
Being pregnant upon arrest (ref: no)						
Yes	0.41***	(0.27, 0.63)	0.37***	(0.24, 0.57)	0.34***	(0.22, 0.52)
Schedule II drugs involvement (ref: two or more)						
One only	1.10	(0.62, 1.93)			1.04	(0.57, 1.91)
Schedule I drug involvement (ref: yes)						
No	0.36***	(0.28, 0.46)			0.48***	(0.36, 0.63)
Drug offence other than use (ref: no)						
Yes	0.80	(0.56, 1.15)			0.56**	(0.38, 0.83)
Type of drug offence in prior 5 years (ref: both)						
Drug use only	0.33***	(0.27, 0.41)			0.35***	(0.29, 0.43)
Other than drug use	0.20***	(0.14, 0.30)			0.23***	(0.16, 0.34)

^a^Multinomial regression model, with awaiting decision/execution (reference), deferred prosecution, correctional rehabilitation, and incarceration as four outcome responses

^b^aOR: adjusted odds ratio

^c^Model I presents the estimates, one by one, with adjustment for drug offence characteristics

^d^Model II includes all listed sociodemographic, motherhood, and drug offence characteristics

^*^p < 0.05, **p < 0 .01, ***p < 0.001

## Discussion

To our knowledge, ours is one of the first studies investigating socioeconomic and motherhood characteristics with regard to prosecutorial decision among drug-using young women involved with the criminal justice system [39]. This population-based data linkage study found that the occurrence of disadvantaged socioeconomic conditions and motherhood experiences were more prevalent among recidivists. In a society where deferred prosecution was the only opportunity for diverting justice-involved drug users into community-based treatment, only one in five first-time offenders and one in sixteen recidivists received deferred prosecution within the first six months of arrest. For first-time drug offenders, low/unstable income is linked with a reduced odds of receiving deferred prosecution. For recidivists, women in worse-off socioeconomic conditions were twice as likely to receive correction-based rehabilitation and incarceration; being pregnant upon arrest was found to lower the odds of receiving a prosecutorial decision, particularly deferred prosecution.

In the study context in which arrested drug users were considered as both criminals and patients, these data demonstrate that the majority (56% of first-time offenders and 59% of recidivists) remained awaiting prosecutorial decisions by the end of the sixth month after arrest. The Department of Justice worked to implement the amendment of Article 24 in the Narcotics Hazard Prevention Act in 2008 [33], particularly launching a comprehensive course to enhance prosecutors’ knowledge of the requirements for reaching deferred prosecution agreements with drug offenders. The policy was also included in pre-service training and on-the-job training through periodic seminars and workshops in which addiction professionals and experienced prosecutors shared their expertise about prosecutorial decisions and prosecution disposition examples. Nonetheless, the implementation of deferred prosecution is hindered or delayed by several constraints, such as excessive prosecutorial caseloads, limited healthcare capacity, and lack of collaboration between justice, healthcare, and public health systems [40].

It is regrettable that only one-fifth of first-time offenders received deferred prosecution within 180 days after arrest. From a public health perspective, early engagement in evidence-based treatment services not only prevents drug users from transitioning into advanced stages of problems, but also reduces health and social harm, which is especially critical for young people [41]. The observed gap in access to treatment in the community may represent a missed opportunity to deliver the indicated intervention for drug problems while avoiding social exclusion. Finally, although the history of drug offences may not linearly reflect on addiction severity or treatment needs, this situation indicates that high-risk, high-need women have a lower chance of receiving evidence-based medical treatment in the community [39, 42]. Whether to treat drug users primarily as criminals (with punishment) or as patients (with treatment) may increase conflicts and dilemmas in prioritizing resources.

Given that treatment-seeking is driven mainly by legal social control, deferred prosecution can admittedly be leveraged as an opportunity to connect drug-using young women with appropriate substance-use treatment in the community [34]. Our analyses show that first-time drug offenders with stable/low income were less likely to receive deferred prosecution. Several mechanisms may be responsible for this socioeconomic status-related disparity, including (1) an inability to afford lawyers’ fees, court-ordered fines, and treatment [43, 44]; (2) the chance or feasibility to take unpaid leave to attend treatment is limited; and (3) the periodic check-ups (including urine tests) required by one-year monitorship by the district prosecutor’s office may restrain freedom of movement and job opportunities. Meanwhile, the association linking worse-off socioeconomic backgrounds with increased probability of receiving correctional rehabilitation and incarceration among the recidivists was dismaying because imprisonment often aggravates drug offenders’ drug problems, social marginalization, and social inequality in this already disadvantaged group [45]. Our results indicate a need for further investigation of the processes underlying this observation, particularly among young women with unfavorable socieoconomic conditions.

Previous studies have indicated that children are often the primary motivation for women to seek or maintain treatment (e.g., concern for the baby’s health or custody of children) [46–48], yet paradoxically, childcare was consistently ranked as the major barrier for their compliance with treatment (e.g., lack of childcare, fear of loss of custody, and stigma) [48–52]. Although this did not reach statistical significance in the final model, we did notice that for justice-involved drug-using young women, childrearing may potentially emerge as a barrier to accessing evidence-based medical treatment in the community. This may be the mixed result of (1) drug-using young women having been disproportionately affected by poor family support and impoverished social capital; (2) addiction treatment services being offered only from 9 a.m. to 5 p.m. Monday to Friday, making access especially challenging for certain casual laborers or homemakers; and (3) childcare in the community being limited, such as to drop-in daycare at court, or lacking [22, 39, 40].

Women who use illegal drugs are a subgroup underrepresented in the healthcare-seeking population. In Taiwan, although no law specifically punishes women using drugs during pregnancy or having a positive test for drugs while giving birth to a baby, service underutilization was unanimously found in prenatal and postnatal care [53, 54]. Conceptually, deferred prosecution is the alternative to incarceration that can facilitate access and engagement in addiction treatment, health care (e.g., prenatal care), and social services (when needed) in communities. Nonetheless, our findings indicate that once arrested as recidivists, expectant mothers will be least likely to receive any service whatsoever, either community or correction based. This observation may result from complex interactions between the following factors: (1) prosecutors’ lack of awareness of drug-using women’s need for health and social care; (2) inadequacy of addiction services or health-justice collaboration networks for pregnant women in the district; and (3) female offenders’ negative perception of treatment, prior experience with healthcare providers, and the support they would gain in community-based treatment [55]. Finally, although the process to reach and implement prosecutorial decisions may depend on an array of offender-, prosecutor-, and region-level factors, the lengthy associated waiting period may undeniably erode external motivation for seeking integrated treatment in the community and even reflect a missed opportunity to intervene against in-uterus drug exposure or unfavorable pregnancy outcomes [12, 56].

The strengths of this study primarily lie in the data linkage between national criminal justice data and healthcare data. Ours is one of the few studies that highlight how socioeconomic and motherhood characteristics may play a role in shaping the chance to receive community- and correction-based treatment among young women arrested for the use of illegal drugs. However, some limitations need to be considered when interpreting our findings. First, our measure of childrearing was a composite indicator ascertained from the birth history; it is unclear how well it reflects the household living arrangement or custody status of the arrested women. Second, due to the protection of minors, the imprisonment records relevant to drug offences (particularly Schedule I/II) before the age of 18 were not ascertained; thus, prior experience of illegal drug use may be underestimated, which is particularly true for those under the age of 24. Third, this study was not designed to assess prosecution mechanisms. Although adjustment has been made based on drug-offender-level characteristics, we cannot rule out that other factors, particularly those arising from the macro-level social environment (e.g., the resources of addiction services available in judiciary districts), may partly explain the observed association of interest. Finally, the associations reported here are contingent upon population characteristics (e.g., age and drug of primary involvement), drug laws, law enforcement, and healthcare systems; generalization to other population subgroups, countries, and societies may be restrained.

## Conclusion

In summary, our analyses show that among justice-involved young women who use illegal drugs like amphetamine-like stimulants, the opportunity of receiving deferred prosecution was significantly increased when the first-time drug offenders had better-off socioeconomic backgrounds; being pregnant upon arrest was associated with reduced opportunity of receiving deferred prosecution for the recidivists. Given that a public health approach has been widely recommended to tackle drug use and associated problems [11, 57], prosecution policy should be informed by female offenders’ need for evidence-based treatment and recovery options [58], particularly when they are rearing a child or pregnant. Further analyses involving and comparing the data from different countries will be needed to confirm how the prosecution process and decision may affect addiction recovery in women [59].

### Supplementary Information

**Additional file 1: Appendix Table 1.** Characteristics in relation to the time to receive deferred prosecution within the year of arrest in the first-time offenders (n = 2645).** Appendix Table 2.** Characteristics in relation to the time to receive deferred prosecution within the year of arrest in the recidivistic offenders (n = 3224).

## Data Availability

The data supporting the findings are obtained from the Department of Statistics, Ministry of Health and Welfare, Taiwan, upon application. All the data were stored in the Health and Welfare Data Science Center. These data are not publicly available due to legal restrictions. Those who wish to request information regarding data from this study, please contact chuanychen@nycu.edu.tw.

## Abbreviations

aOR: Adjusted odds ratio
BRS: Birth Reporting System
CI: Confidence interval
DALYs: Disability-adjusted life-years
DPCCAT: Deferred Prosecution with Condition to Complete the Addiction Treatment
ICD-9-CM: International Classification of Diseases, Ninth Revision, Clinical Modification
MDMA: 3,4-Methylenedioxymethamphetamine
OR: Odds ratio
