# Supplementary material for: Association between socioeconomic and motherhood characteristics with receiving community-based treatment services among justice-involved young female drug users: a retrospective cohort study in Taiwan
Source: Harm Reduct J. 2024 Jun 5;21:109. doi: 10.1186/s12954-024-01010-0 (PMC11151603; doi:10.1186/s12954-024-01010-0)
Supplement: Supplementary file 1 — Additional file 1: Appendix Table 1. Characteristics in relation to the time to receive deferred prosecution within the year of arrest in the first-time offenders (n = 2645). Appendix Table 2. Characteristics in relation to the time to receive deferred prosecution within the year of arrest in the recidivistic offenders (n = 3224). [file 12954_2024_1010_MOESM1_ESM.docx]

**Supplementary materials**

**Appendix Table 1**

Characteristics in relation to the time to receive deferred prosecution within the year of arrest in the first-time offenders (n=2,645)^a^

| **Variables** | **Univariate** | | | **Model I^b^** | | | |
| --- | --- | --- | --- | --- | --- | --- | --- |
|  | ***HR*** | ***95% CI*** | | ***aHR*** | | ***95% CI*** | |
| Age (ref: 25-29) |  |  |  |  | |  |  |
| 18-24 | 1.04 | (0.88 | ,1.20) | | 0.93 | (0.79 | ,1.11) |
| Marital status (ref: single) |  |  |  | |  |  |  |
| Married | 0.90 | (0.71 | ,1.14) | | 0.97 | (0.74 | ,1.27) |
| Divorced or widowed | 0.89 | (0.72 | ,1.11) | | 0.97 | (0.75 | ,1.24) |
| Educational attainment (ref: senior high school or above) |  |  |  | |  |  |  |
| Junior high school | 0.95 | (0.81 | ,1.12) | | 0.99 | (0.83 | ,1.17) |
| Employment status (ref: employed) |  |  |  | |  |  |  |
| Unemployed/student | 0.88 | (0.76 | ,1.03) | | 0.89 | (0.76 | ,1.04) |
| Income level (ref: medium/high income) |  |  |  | |  |  |  |
| Low/unstable income | 0.82^*^ | (0.70 | ,0.95) | | 0.84^*^ | (0.72 | ,0.98) |
| Having one or more young children (ref: no) |  |  |  | |  |  |  |
| Yes | 0.85 | (0.69 | ,1.04) | | 0.89 | (0.70 | ,1.12) |
| Being pregnant upon arrest (ref: no) |  |  |  | |  |  |  |
| Yes | 0.95 | (0.66 | ,1.38) | | 1.00 | (0.69 | ,1.47) |
| Scheduled II drugs involvement (ref: two or more) |  |  |  | |  |  |  |
| One only | 0.92 | (0.70 | ,1.82) | | 0.86 | (0.56 | ,1.33) |
| Scheduled I drug involvement (ref: yes) |  |  |  | |  |  |  |
| No | 1.14 | (0.55 | ,1.43) | | 0.95 | (0.58 | ,1.56) |
| Drug offence other than use (ref: no) |  |  |  | |  |  |  |
| Yes | 0.44^***^ | (0.30 | ,0.66) | | 0.44^***^ | (0.29 | ,0.65) |
| Drug offence more than five years (ref: yes) |  |  |  | |  |  |  |
| No | 1.52^*^ | (1.01 | ,2.27) | | 1.56^*^ | (1.02 | ,2.38) |

*^*^ p < 0.05, ^**^p <0.01, ^***^p<0.001*

1. Cox proportional regression model, the outcome event was receiving deferred prosecution within the year of arrest, adjusted hazard ratio (aHR)
2. Model I adjusted for sociodemographics, drug offence characteristics (drug type and nondrug use involvement), and history of drug offence during adulthood.

**Appendix Table 2**

Characteristics in relation to the time to receive deferred prosecution within the year of arrest in the recidivistic offenders (N=3,224)^a^

| **Variables** | **Univariate** | | | | **Model I^b^** | | | |
| --- | --- | --- | --- | --- | --- | --- | --- | --- |
|  | ***HR*** | | ***95% CI*** | | ***aHR*** | | ***95% CI*** | |
| Age (ref: 25-29) |  | |  |  |  | |  |  |
| 18-24 | | 1.37^*^ | (1.06 | ,1.75) | | 1.28 | (0.98 | ,1.69) |
| Marital status (ref: single) | |  |  |  | |  |  |  |
| Married | | 0.79 | (0.55 | ,1.13) | | 1.10 | (0.74 | ,1.63) |
| Divorced or widowed | | 0.78 | (0.56 | ,1.10) | | 1.02 | (0.70 | ,1.49) |
| Educational attainment (ref: senior high school or above) | |  |  |  | |  |  |  |
| Junior high school | | 0.85 | (0.63 | ,1.13) | | 0.85 | (0.63 | ,1.15) |
| Employment status (ref: employed) | |  |  |  | |  |  |  |
| Unemployed/student | | 0.96 | (0.74 | ,1.23) | | 1.04 | (0.81 | ,1.35) |
| Income level (ref: medium/high income) | |  |  |  | |  |  |  |
| Low/unstable income | | 0.74^*^ | (0.56 | ,0.99) | | 0.87 | (0.65 | ,1.16) |
| Having one or more young children (ref: no) | |  |  |  | |  |  |  |
| Yes | | 0.64^**^ | (0.47 | ,0.88) | | 0.70^*^ | (0.49 | 1.00) |
| Being pregnant upon arrest (ref: no) | |  |  |  | |  |  |  |
| Yes | | 0.39^**^ | (0.20 | ,0.76) | | 0.43^*^ | (0.22 | ,0.85) |
| Scheduled II drugs involvement (ref: two or more) | |  |  |  | |  |  |  |
| One only | | 0.80 | (0.40 | ,1.61) | | 0.84 | (0.41 | ,1.69) |
| Scheduled I drug involvement (ref: yes) | |  |  |  | |  |  |  |
| No | | 1.33 | (0.81 | ,2.17) | | 1.05 | (0.63 | ,1.75) |
| Drug offence other than use (ref: no) | |  |  |  | |  |  |  |
| Yes | | 0.92 | (0.56 | ,1.50) | | 1.02 | (0.62 | ,1.69) |
| Type of drug offence in prior 5 years (ref: drug use only) | |  |  |  | |  |  |  |
| Other than drug use | | 1.08 | (0.77 | ,1.50) | | 1.04 | (0.74 | ,1.45) |
| Both | | 0.55^***^ | (0.40 | ,0.74) | | 0.59^***^ | (0.43 | ,0.80) |

*^*^ p < 0.05, ^**^p <0.01, ^***^p<0.001*

1. Cox proportional regression model, the outcome event was receiving deferred prosecution within the year of arrest and adjusted hazard ratio (aHR).
2. Model I adjusted for sociodemographic characteristics, characteristics of the index arrest, and past drug offence characteristics.
